# Efficacy of a six‐portal arthroscopic technique in managing acute septic arthritis of the native knee: A retrospective cohort study in a tertiary orthopaedic department

**DOI:** 10.1002/jeo2.70866

**Published:** 2026-07-20

**Authors:** Efstathios Konstantinou, Theodorakys Marín Fermín, Georgios Kalifis, Nikolaos Stefanou, Georgios Komnos, Michael Hantes

**Affiliations:** ^1^ Department of Orthopaedic Surgery and Musculoskeletal Trauma University Hospital of Larissa, School of Health Sciences, University of Thessaly Larissa Greece; ^2^ Clínica Santa Sofía Caracas Venezuela; ^3^ Thessaloniki Minimally Invasive Surgery Orthopaedic Center St. Luke's Hospital Panorama Greece

**Keywords:** arthroscopy, clinical outcomes, native knee, septic arthritis, synovectomy

## Abstract

**Purpose:**

To evaluate the clinical outcomes of a six‐portal arthroscopic technique for the management of acute septic arthritis of the native knee in a tertiary orthopaedic department.

**Methods:**

A retrospective cohort study was conducted, including patients with acute septic arthritis of the knee treated with a six‐portal arthroscopic debridement technique between 2017 and 2023. All patients had a minimum follow‐up of 12 months. Clinical and functional outcomes were assessed using the Knee Injury and Osteoarthritis Outcome Score (KOOS), International Knee Documentation Committee (IKDC) score, and Tegner–Lysholm score. Additional parameters included microbiological findings, hospital stay, and recurrence or reoperation rates.

**Results:**

Twenty‐eight patients were included, with a mean age of 52 years (range: 32–72) and a mean follow‐up of 45.4 months (range: 12–72). The mean hospital stay was 12.1 days (range: 7–20). *Staphylococcus* species were identified as the most common causative organisms (43%). No recurrence of infection or need for revision surgery was observed during the follow‐up period. At final evaluation, functional outcomes demonstrated a mean KOOS of 80 (range: 64–92), a mean IKDC score of 74.5 (range: 61–86) and a mean Tegner–Lysholm score of 84.7 (range: 70–95), indicating overall good knee function and consistent clinical improvement across the cohort.

**Conclusion:**

The six‐portal arthroscopic technique demonstrated favourable clinical outcomes in the management of acute septic arthritis of the native knee, with no recurrence or need for repeat debridement observed in this cohort. While these findings are encouraging, further comparative and higher‐volume studies are required to determine whether this approach offers advantages over standard arthroscopic techniques.

**Level of Evidence:**

Level IV.

**Abbreviations**
IKDC: International Knee Documentation Committee
KOOS: Knee Injury and Osteoarthritis Outcome Score
SD: standard deviation

## INTRODUCTION

Septic arthritis is an orthopaedic emergency requiring prompt diagnosis and intervention to avoid severe complications [9, 11]. The knee is the most commonly affected joint, accounting for approximately 50% of cases, with *Staphylococcus aureus* as the predominant pathogen [19, 29]. The incidence of bacterial joint infection in Western Europe ranges from 4 to 10 per 100,000 individuals annually and is rising [4]. Although uncommon, acute septic arthritis carries substantial risk, particularly in patients with underlying joint disease, prosthetic joints, or comorbidities such as diabetes and renal failure [9, 15]. Potential complications include irreversible joint destruction, osteomyelitis, septicaemia and a 30‐day mortality rate of 1%–9%, reaching nearly 10% in some cohorts [6, 10, 20]. The management of septic arthritis aims to achieve complete eradication of the infection through antibiotics and aggressive surgical debridement [20]. Prompt initiation of intravenous antibiotics after joint aspiration is essential, and empirical antibiotic therapy is guided by local institutional protocols [3, 12].

Surgical treatment may involve open arthrotomy or arthroscopic debridement [20]. Although arthroscopy is minimally invasive and provides improved visualization, its superiority over arthrotomy remains debated [1, 17, 20]. Recent meta‐analyses report conflicting results; some demonstrate lower re‐infection rates and faster recovery with arthroscopy, whereas others show no significant difference [1, 17, 20]. Functional and clinical outcomes also remain uncertain, partly due to heterogeneity in patient characteristics and disease severity across published studies [20].

Arthroscopic irrigation and debridement are widely regarded as optimal due to the minimally invasive approach and the potentially lower complication rates [1, 20]. However, the standard two‐portal anterior arthroscopy has limitations, particularly in accessing and adequately debriding the posterior recesses of the knee, which may harbour residual organisms and compromise eradication of infection [18]. To address these challenges, a six‐portal arthroscopic technique has been proposed, enabling a more complete synovectomy and lavage of all joint compartments, including the posterior recesses. Although initially described in recalcitrant cases [18], evidence assessing its clinical outcomes in primary acute septic arthritis remains limited.

The aim of this study was to evaluate the efficacy and outcomes of a six‐portal arthroscopic technique for treating acute septic arthritis of the native knee in a tertiary orthopaedic department. We hypothesized that this technique would result in satisfactory clinical outcomes with minimal need for subsequent debridement.

## MATERIAL AND METHODS

### Study design

A retrospective cohort study was conducted at our institution, including all consecutive cases of acute septic arthritis of the native knee managed with a six‐portal arthroscopic debridement technique between July 2017 and June 2023.

### Study participants and eligibility criteria

Patients were included if they had a confirmed diagnosis of acute septic knee arthritis, underwent the six‐portal arthroscopic technique and had a minimum of 12 months of postoperative follow‐up. Diagnosis was established based on the patient's clinical presentation, laboratory findings, including inflammatory markers and synovial fluid analysis, and microbiological confirmation where available. Knee aspiration was performed prior to any intervention. Synovial fluid leukocyte count was interpreted as one component of the diagnostic work‐up together with the clinical presentation, inflammatory markers, synovial fluid appearance and microbiological findings. The decision to proceed with urgent arthroscopic debridement was based on the overall clinical assessment rather than on a predefined synovial leukocyte threshold, in accordance with current recommendations [24]. Exclusion criteria included chronic septic arthritis, prosthetic joint infections, any other prior operation on the affected knee, or antibiotic use before the arthroscopic debridement. Two authors (E. K. and G. K.) independently identified eligible cases through a computerized surgical database and reviewed the medical records to extract preoperative, intraoperative, and postoperative data.

### Surgical technique

The six‐portal arthroscopic technique involved the strategic placement of anteromedial, anterolateral, superomedial, superolateral, posteromedial and posterolateral portals to ensure optimal joint visualization and access. Particular attention was given to the posterior compartments, where thorough synovectomy and lavage were performed through posteromedial and posterolateral portals. The procedure was performed systematically to allow circumferential synovectomy of the knee, addressing the anterior, posterior, medial, and lateral compartments, as well as the suprapatellar pouch and both medial and lateral recesses. This approach was intended to facilitate thorough irrigation and removal of purulent material from all joint compartments, including the posterior recesses. All operations were performed by the senior surgeon (M. H.). Gächter's classification was recorded during the arthroscopy [8]. Postoperative joint drainage was performed using suction catheters for 48 h. Antibiotic therapy was initiated empirically postoperatively and adjusted based on culture sensitivity results by the hospital's Department of Infectious Diseases.

### Postoperative protocol

Patients were monitored for signs of infection resolution and restoration of joint function. Empirical intravenous clindamycin was continued until microbiological results identified the causative pathogen. Thereafter, antibiotic therapy was adjusted according to culture and antimicrobial susceptibility results and continued intravenously or orally, as recommended by specialists from the Department of Infectious Diseases. The duration of treatment was individualized according to the identified pathogen, clinical course, and serial inflammatory markers. Early passive and active range‐of‐motion exercises were initiated postoperatively to minimize stiffness; weight‐bearing was permitted as tolerated.

### Outcomes and follow‐up

Clinical and functional outcomes were assessed at a minimum of 12 months postoperatively using validated scoring systems, including the Knee Injury and Osteoarthritis Outcome Score (KOOS), International Knee Documentation Committee (IKDC) score, and Tegner–Lysholm score. Clinical outcomes were interpreted according to previously described thresholds and reference values for each scoring system. The Tegner–Lysholm score was categorized as poor (<65), fair (65–83), good (84–90), and excellent (>90), as previously reported [5]. IKDC and KOOS scores were interpreted according to previously published reference values, with higher scores indicating better knee function [13, 25]. Gächter's classification was also documented as part of the outcome assessment; this arthroscopic staging system grades septic arthritis from Stage I to IV based on synovial inflammation, fibrin deposition, and cartilage or subchondral bone involvement, thereby indicating disease severity [7]. Additional parameters, including microbiological findings, patient comorbidities, hospital stay duration, and recurrence or reoperation rates, were recorded. Radiographic evaluations were performed postoperatively to ensure the absence of structural complications.

## RESULTS

Twenty‐eight patients were included in the study, with 24 men (85.7%) and four women (14.3%). The mean age of the cohort was 52 years (range: 32–72). Comorbidities included diabetes mellitus in 12 patients (43%) and rheumatoid arthritis in four patients (14.3%). A potential source of infection was identified in 18 patients (64%), including urinary tract infections, respiratory infections, and recent invasive procedures. In the remaining 10 patients (36%), no identifiable source could be determined.

The mean postoperative follow‐up was 45.4 months (range: 12–72). The mean hospital stay was 12.1 days (range: 7–20). The Gächter classification revealed that 10 patients (36%) presented with Stage I disease, 14 (50%) with Stage II, and 4 (14%) with Stage III.

Microbiological analysis identified *Staphylococcus* species as the most common causative organisms in 12 cases (43%). Other pathogens included *Streptococcus pyogenes* (6 cases, 22%), *Escherichia coli* (4 cases, 14%), and *Proteus mirabilis*, *Providencia rettgeri,* and *Streptococcus agalactiae*, each accounting for two cases (7%).

Clinical outcomes demonstrated favourable results, with no recurrence of infection or need for revision surgery. Postoperative radiographs were reviewed in all patients and did not reveal any additional clinically relevant findings. Functional scores at the final follow‐up showed a mean KOOS of 80 (standard deviation [SD] 2.8), a mean IKDC score of 74.5 (SD 7.0) and a mean Tegner–Lysholm score of 84.7 (SD 3.5), corresponding overall to good clinical outcomes. These favourable outcomes were observed across all stages of the Gächter classification. All results are summarized in Table 1.

**Table 1:** Summary of clinical and microbiological outcomes.

Demographics	
Male	24 (85.7%)
Female	4 (14.3%)
Mean age (years)	52 (32–72)
Comorbidities	
Diabetes mellitus	12 (43%)
Rheumatoid arthritis	4 (14.3%)
Microbiology	
*Staphylococcus* species	12 (43%)
*Streptococcus pyogenes*	6 (22%)
*Escherichia coli*	4 (14%)
*Proteus mirabilis*	2 (7%)
*Providencia rettgeri*	2 (7%)
*Streptococcus agalactiae*	2 (7%)
Hospital stay (days)	
Days	Mean 12.1 (range: 7–20)
Gächter classification	
Stage I	10 (36%)
Stage II	14 (50%)
Stage III	4 (14%)
Functional scores	
KOOS	Mean 80 (range: 64–92)
IKDC	Mean 74.5 (range: 61–86)
Tegner–Lysholm	Mean 84.7 (range: 70–95)

Abbreviations: IKDC, International Knee Documentation Committee; KOOS, Knee Injury and Osteoarthritis Outcome Score.

No surgical complications were observed in this cohort, including neurovascular injury, iatrogenic cartilage damage, or compartment syndrome. Additionally, no major systemic complications such as thromboembolic events or sepsis were recorded. Overall, seven patients (25%) developed minor medical complications, including five mild allergic reactions and two cases of pseudomembranous colitis, all of which were managed successfully by the Department of Infectious Diseases without further surgical intervention.

## DISCUSSION

The main finding of this retrospective study is that the six‐portal arthroscopic technique for treating acute septic arthritis of the native knee is an effective option, yielding favourable patient‐reported outcomes without the need for repeat debridement, regardless of Gächter classification, at a minimum 1‐year follow‐up, thereby supporting our hypothesis.

Recent systematic reviews on native knee septic arthritis have shown similar outcomes between open and arthroscopic treatment [16, 21, 23]. McKenna et al. [20], in a study pooling data from nine articles including 17,140 patients, reported no statistical differences in the overall rate of repeat washout (14.6%, risk ratio 0.86) or mortality rate (risk ratio 1.17), while favouring arthroscopy in terms of all‐cause complications (risk ratio 0.75) and hospital stay (mean difference −1.98 days). Similarly, Nudelman et al. [21] found no significant differences in reoperation rates in large and intermediate‐sized joints across 14 included studies, although clinical outcomes tended to favour arthroscopic treatment.

Another systematic review by Kennedy et al. [16], including seven articles, found a slight trend favouring arthroscopic washout over open treatment, although this was unlikely to be clinically relevant in most cases. The heterogeneity of patient‐reported outcomes in studies assessing native knee septic arthritis makes direct comparisons with our results difficult, particularly given differences in study design, patient populations and outcome measures [2, 22, 26, 30].

Given the lack of comparative studies on arthroscopic techniques for managing native knee septic arthritis, the presented six‐portal arthroscopic technique shows promising outcomes, with no reoperations or infection recurrence observed in our cohort at a mean follow‐up of 45.4 months, compared with the cited systematic reviews. This may be explained by the comprehensive access afforded by the six‐portal technique, which facilitates visualization and debridement of all knee compartments, including the posterior recesses. However, in the absence of a comparative cohort, it cannot be determined whether this translates into superior clinical outcomes compared with conventional arthroscopic techniques. This approach is consistent with previously described concepts of complete arthroscopic synovectomy, in which thorough debridement of all compartments is considered essential to reduce the bacterial load and minimize the need for repeat procedures. Although previous studies have generally reported shorter hospital stays following arthroscopic treatment of septic arthritis [27, 28], the duration of hospitalization is influenced by several factors beyond the surgical approach itself. In our institution, discharge is determined jointly with the Department of Infectious Diseases once patients are clinically stable and an appropriate antimicrobial regimen has been established. Furthermore, the relatively high prevalence of medical comorbidities in our cohort may also have contributed to the observed length of stay. Microbiological analysis identified *Staphylococcus* species (*S. aureus* and *S. epidermidis*) and *Streptococcus pyogenes* as the most common causative organisms, consistent with previous reports [14, 22].

This study has several limitations, including its retrospective design, a relatively small patient cohort, and the lack of a control group. The absence of detailed data on antibiotic therapy limits the assessment of the role of multidisciplinary management in this setting. Furthermore, as a single surgeon performed all procedures, the technique's reproducibility and associated learning curve warrant further evaluation in future studies. Additionally, variability in the timing of presentation and severity of infection at diagnosis may have influenced outcomes. The absence of a comparison group treated with standard arthroscopic techniques also limits the ability to determine whether the observed outcomes are attributable specifically to the six‐portal approach. Conversely, one of the strengths of this study is the use of validated outcome measures, including KOOS, IKDC and Tegner–Lysholm scores, allowing for meaningful comparison with existing literature.

## CONCLUSION

The six‐portal arthroscopic technique demonstrated favourable clinical outcomes in the management of acute septic arthritis of the native knee, with no recurrence or need for repeat debridement observed in this cohort. While these findings are encouraging, further comparative and higher‐volume studies are required to determine whether this approach offers advantages over standard arthroscopic techniques.

## AUTHOR CONTRIBUTIONS

All listed authors have made substantial contributions to this work. Literature search, study design, and primary manuscript preparation were performed by Efstathios Konstantinou and Theodorakys Marín Fermín. Data collection, analysis, and interpretation of the results were performed by Efstathios Konstantinou and Georgios Kalifis. Statistical analysis was performed by Efstathios Konstantinou. Final manuscript drafting and critical revision were performed by Theodorakys Marín Fermín, Nikolaos Stefanou, Georgios Komnos, and Michael Hantes. All authors have read and approved the final version of the manuscript.

## FUNDING INFORMATION

The authors have no funding to report.

## CONFLICT OF INTEREST STATEMENT

The authors declare no conflict of interest.

## ETHICS STATEMENT

Institutional review board approval was obtained prior to study initiation from the Institutional Review Board of University Hospital of Larissa, Greece (approval number: 48188/07‐11‐25). The study was conducted in accordance with the principles of the Declaration of Helsinki. Patient consent was waived due to the retrospective nature of the study.

## Data Availability

Anonymised data from the study are available upon reasonable request.
